# Association between polymorphism rs12722 in COL5A1 and musculoskeletal soft tissue injuries: a systematic review and meta-analysis

**DOI:** 10.18632/oncotarget.23805

**Published:** 2017-12-27

**Authors:** Zheng-Tao Lv, Shu-Tao Gao, Peng Cheng, Shuang Liang, Si-Yi Yu, Qing Yang, An-Min Chen

**Affiliations:** ^1^ Department of Orthopedics, Tongji Hospital, Tongji Medical College, Huazhong University of Science and Technology, Wuhan, Hubei 430030, China; ^2^ Department of Spine Surgery, The First Affiliate Hospital of Xinjiang Medical University, Urumqi, Xinjiang 830054, China; ^3^ The 3rd Teaching Hospital, Chengdu University of Traditional Chinese Medicine, Chengdu 610075, China

**Keywords:** COL5A1, polymorphism, rs12722, tendon and ligament injury, systematic review

## Abstract

The rs12722 polymorphism in COL5A1 gene has been implicated in the etiology of musculoskeletal soft tissue injuries in several association studies with limited sample size and conflicting results. The purpose of the present systematic review and meta-analysis was to evaluate and synthesize the currently available data on the association between rs12722 and musculoskeletal soft tissue injuries. Five electronic databases including Pubmed, EMBASE, ISI Web of Science, CNKI and Wanfang were searched to identify relevant studies published before 15 May, 2017. Summary odds ratios (ORs) and corresponding 95% confidence intervals (95% CIs) were estimated using the RevMan 5.3 software. Nine studies comprising 1140 cases and 1410 healthy controls met the eligibility criteria. Recessive model was confirmed to be the optimum model (TT vs TC + CC). The results indicated that rs12722 SNP was significantly associated with musculoskeletal soft tissue injuries (OR 1.58, 95% CI 1.33, 1.89; *P* < 0.00001). When stratified by injury sites, modest but statistically significant association was found in Achilles tendon pathology (ATP), anterior cruciate ligament injuries (ACLI) and tennis elbow (TE). Subgroup-analysis by ethnicity suggested that TT genotype of rs12722 was associated with tendon and ligament injuries in Caucasians (OR 1.59, 95% CI 1.33, 1.90; *P* < 0.00001) but not in Asians (OR 1.46, 95% CI 0.46, 4.60; *P* = 0.52). Our findings indicated that rs12722 of COL5A1 was positively associated with tendon and ligament injuries, especially in Caucasian subjects. Individuals with TT genotype were predisposed to higher risk of ATP, ACLI and TE.

## INTRODUCTION

Tendons and ligaments, both representing dense connective tissues and having comparably structural and mechanical properties, are essential constructions for the musculoskeletal system. The major duty of tendons is to deliver force from muscles to skeletons, causing the connected joints in locomotion, whereas the general function of ligaments is to stabilize joints and guide joints through their normal range of motion [1, 2]. Typical musculoskeletal soft tissue injuries including anterior cruciate ligament (ACL) injuries, Achilles tendon pathology (ATP) [3], tennis elbow, together with rotator cuff tendon injuries usually occur to individuals who endure large amounts of exercise. Of these injuries, ACL injuries and ATP injuries occupy the predominant number [3, 4]. Epidemiological studies have indicated that the incidence of both tendon and ligament injuries are in an increasing trend [5–8]. The causations of tendon and ligament injuries are habitually considered to be either acute traumatic incidences or chronic overburdened activities. Break of the anterior cruciate ligament and Achilles tendon are cases of acute injuries, while Achilles tendinopathy and tennis elbow are examples of chronic overuse injuries.

Other than the two commonly accepted extrinsic causations, intrinsic factors such as genetic factors in conjunction with the gender, biomechanical, anatomic, hormonal, neuromuscular factors are as well supposed to contribute greatly to these injuries [9–12]. Collagens, the foremost elements of connective tissues and extracellular matrix, are the most abundant protein in tendons and ligaments and comprise around 75% of their dry weight [2]. Numerous diseases have been identified caused by mutations in genes for collagens or collagen-processing enzymes [13]. Type V collagen, which is a quantitatively minor fibrillar collagen with an extensive tissue distribution, belongs to the subtype of fibril collagens and normally consists of two α1 polypeptide chain and two α2 polypeptide chains [14]. The α1 chain is encoded by alpha 1 type V collagen (COL5A1) gene. Mokone et al. initially studied the BstUI (rs12722) and DpnII restriction fragment length polymorphisms (RFLPs) in COL5A1 gene and found that BstUI RFLP was strongly associated with ATP [15]. Since then, multiple genetic single nucleotide polymorphisms (SNPs) of COL5A1 [15], COL1A1 [16], COL12A1 and COL14A1 [17], COL27A1 [18], COL11A1 and COL11A2 [19], COL3A1 and COL6A1 [20] have to date been successively studied for the association with the susceptibility of either tendon or ligament injuries. Of these, the COL5A1 SNPs are most frequently studied.

Up to now, several studies focusing on the rs12722 SNP and the susceptibility of tendon and ligament injuries in separate population have been reported, but the sample size of individual study is limited and the findings were inconsistent. Thus, we conducted this systematic review and meta-analysis to investigate more accurate association between rs12722 SNP and tendon and ligament injuries.

## RESULTS

### Literature search and study characteristics

**Figure 1:**
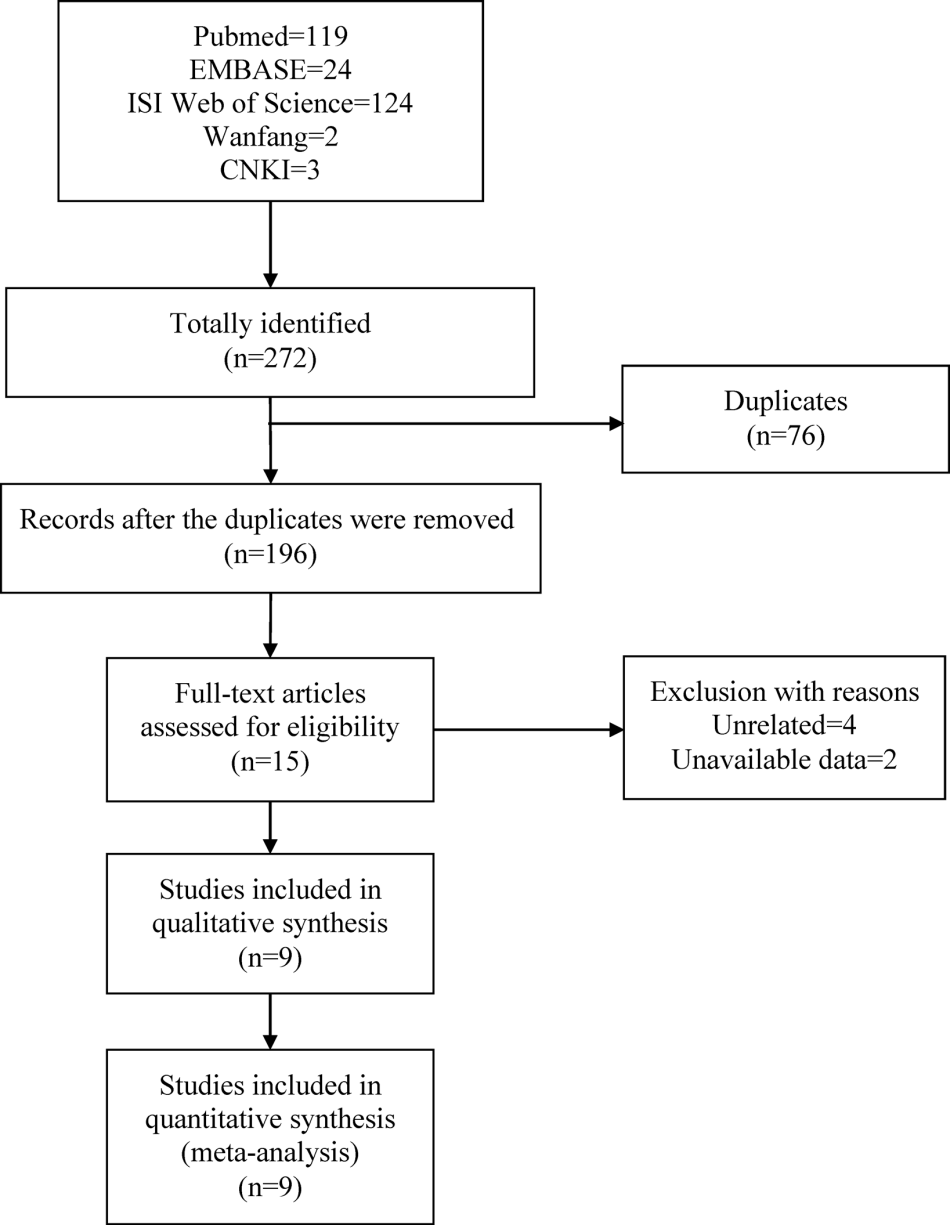
Flow chart of literature selection

A total of 272 potentially relevant articles were identified by the initial literature search, including 119 from Pubmed, 24 from EMBASE, 124 from ISI Web of Science, 2 from Wanfang and 3 from CNKI. After the removal of 76 duplicated records, 196 studies were subsequently screened for eligibility with abstracts and titles. 181 records were removed because of irrelevance. The full-text of fifteen potentially related studies were downloaded to confirm eligibility, four of which were excluded for irrelevance, two studies were excluded due to unavailable data. Finally, nine case-control studies on rs12722 in COL5A1 gene were deemed eligible for inclusion in this met-analysis. The literature screen process was presented in Figure 1.

Nine case-control studies [15, 20–27] comprising a total of 1140 cases and 1410 healthy controls were included for meta-analysis. All these studies were performed in a single center and published from 2006 to 2017. Four studies [15, 20, 25, 26] were performed in South Africa, the five remaining studies were conducted in South Korea [24], China [23], Poland [27], UK [22] and Turkey [21], respectively. Patients with musculoskeletal soft tissue injuries were enrolled due to tennis elbow, Achilles tendon pathology and ACL injury. All the eligible studies provide genotypes distribution in both case groups and control groups. Except for Mokone et al. [15], all the studies complied with HWE. The main characteristics of included trials were summarized in Table 1.

**Table 1:** Main characteristics of included studies and genotype distribution of rs12722 in cases and controls

Study	Country	Ethnicity	Diagnosis	Case			Control			HWE
				TT	TC	CC	TT	TC	CC	
Altinisik, 2015	Turkey	Caucasian	TE	62	46	37	49	85	50	0.587
Brown, 2015	UK	Caucasian	TEN, RUP	26	46	28	27	51	22	0.858
Chen, 2017	China	Asian	ACLI	7	38	60	4	37	49	0.659
Kim, 2015	South Korea	Asian	ACLI, RUP	0	15	20	0	8	31	0.775
Mokone, 2006	South Africa	Caucasian	TEN, RUP	78	7	16	81	9	34	< 0.001
O’Connell, 2014	South Africa	Caucasian	ACLI	104	165	46	103	189	82	0.965
Posthumus, 2009	South Africa	Caucasian	ACLI	42	68	17	62	110	46	0.977
Raleigh, 2008	South Africa	Caucasian	TEN	29	34	11	25	43	30	0.493
Stepien-Slodkowska, 2015	Poland	Caucasian	ACLI	48	66	24	53	91	39	0.999

TE: tennis elbow; TEN: Achilles tendinopathy; RUP: Achilles tendon rupture; ACLI: anterior cruciate ligament injury.

### Quality assessment

The NOS for non-randomized controlled trials were used to assess the risk of bias in included studies. The results for risk of bias judgement were consistent between two reviewers. Except for Kim et al. [24], all the studies reported adequate definitions of cases. Musculoskeletal soft tissue injuries were diagnosed according to the clinical criteria and confirmed by imaging. However, none of our included studies used random sampling method to recruit participants with musculoskeletal soft tissue injuries, and no study enrolled consecutive patients. Five studies [22–24, 26, 27] used hospital controls, thus the representativeness of controls in relevant studies was insufficient. Regarding “Control for important factor or additional factor”, four studies [15, 21, 22, 25] gained two stars because they matched age and other important confounders. The quality assessment by NOS scores of each individual study were listed in Table 2, with an average of 6.2 stars for each included study.

**Table 2:** Methodological quality of included studies

Item/Study	Altinisik, 2015	Brown, 2015	Chen, 2017	Kim, 2015	Mokone, 2006	O’Connell, 2014	Posthumus, 2009	Raleigh, 2008	Stepien-Slodkowska, 2015
Adequate definition of cases	^*^	^*^	^*^	-	^*^	^*^	^*^	^*^	^*^
Representativeness of cases	-	-	-	-	-	-	-	-	-
Selection of control subjects	^*^	-	-	-	^*^	^*^	^*^	-	-
Definition of control subjects	^*^	^*^	^*^	^*^	^*^	^*^	^*^	^*^	^*^
Control for important factor or additional factor	^**^	^**^	-	-	^**^	-	^**^	-	-
Exposure assessment	^*^	^*^	^*^	^*^	^*^	^*^	^*^	^*^	^*^
Same method of ascertainment for all subjects	^*^	^*^	^*^	^*^	^*^	^*^	^*^	^*^	^*^
Non-response rate	^*^	^*^	^*^	^*^	^*^	^*^	^*^	^*^	^*^

A study could be awarded a maximum of one star for each item except for the item “Control for important factor or additional factor”. The definition/explanation of each column of the Newcastle-Ottawa Scale is available from http://www.ohri.ca/programs/clinical_epidemiology/oxford.asp.

### Meta-analysis and subgroup-analysis

**Figure 2:**
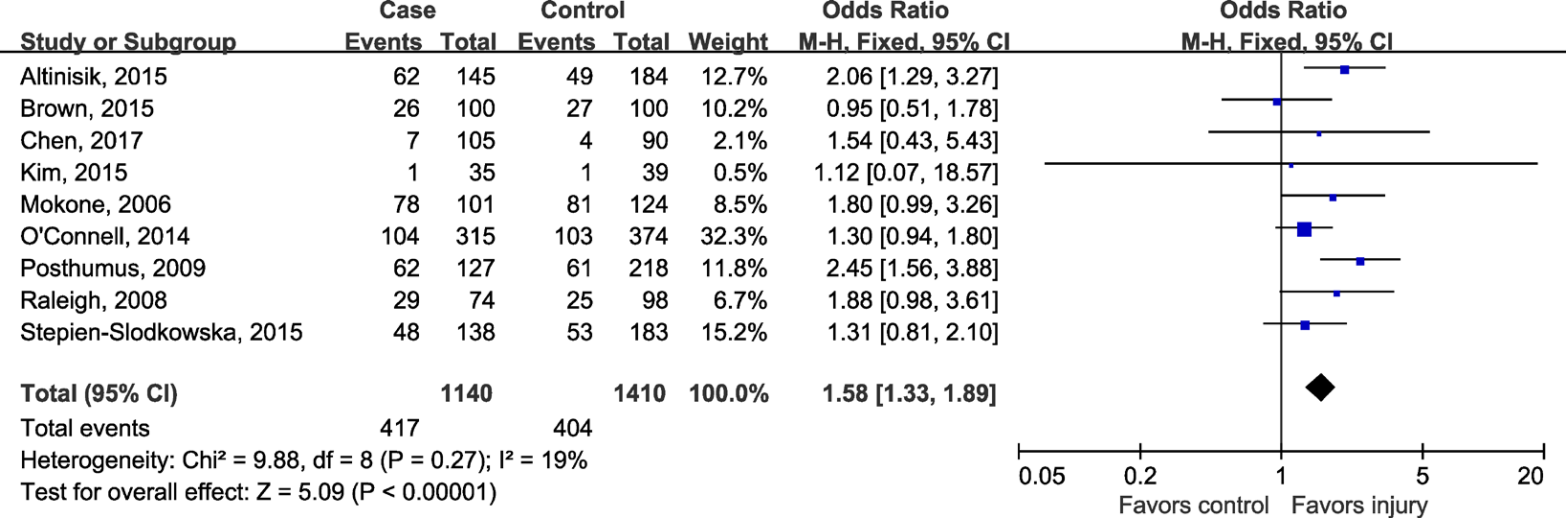
Forest plot of rs12722 for tendon and ligament injuries using a recessive model

Prior to pooling data from individual study, the magnitude of estimate of genetic effect was assessed using the model-free approach as recommended by Thakkinstian [28]. The estimated OR1 (TT/CC: 1.69, 95% CI 1.34, 2.13; *P* < 0.00001) and OR3 (TT/TC: 1.31, 95% CI 1.08, 1.59; *P* = 0.007) turned out to be statistically significant, whereas the OR2 (TC/CC: 1.24, 95% CI 0.92, 1.67; *P* = 0.15) was not significant, suggesting that recessive model could be the most appropriate model for meta-analysis. Using a recessive model, data for TC and CC were collapsed and compared to TT group. Since there was no evidence for between-study heterogeneity (*P* = 0.27, I^2^ = 19%), the combined ORs showed that subjects with TT genotype had a 58 percent higher risk of musculoskeletal soft tissue injuries than people who had TC/CC genotypes (OR 1.58, 95% CI 1.33, 1.89; *P* < 0.00001) (Figure 2).

**Figure 3:**
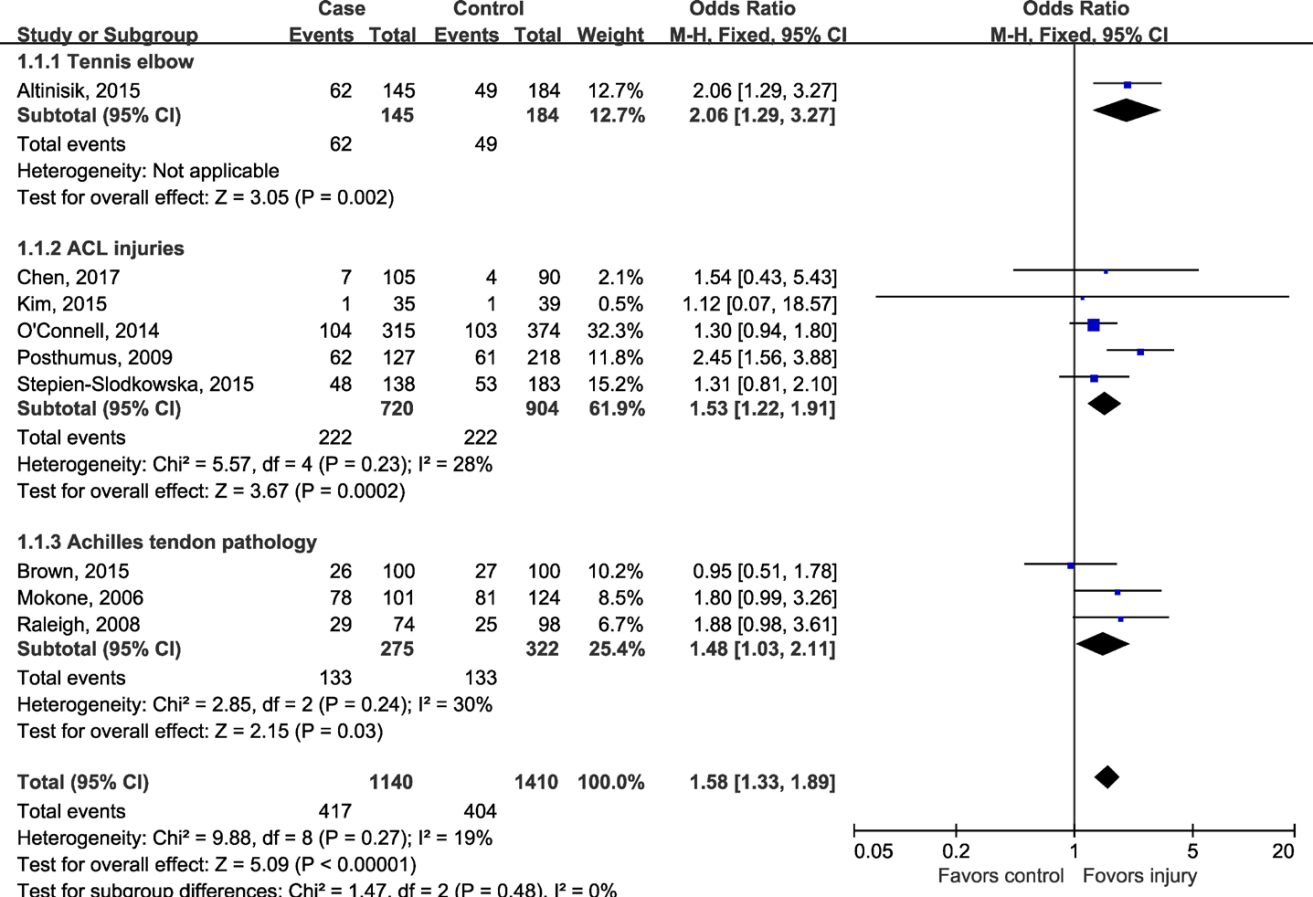
Subgroup-analysis by injury sites

**Figure 4:**
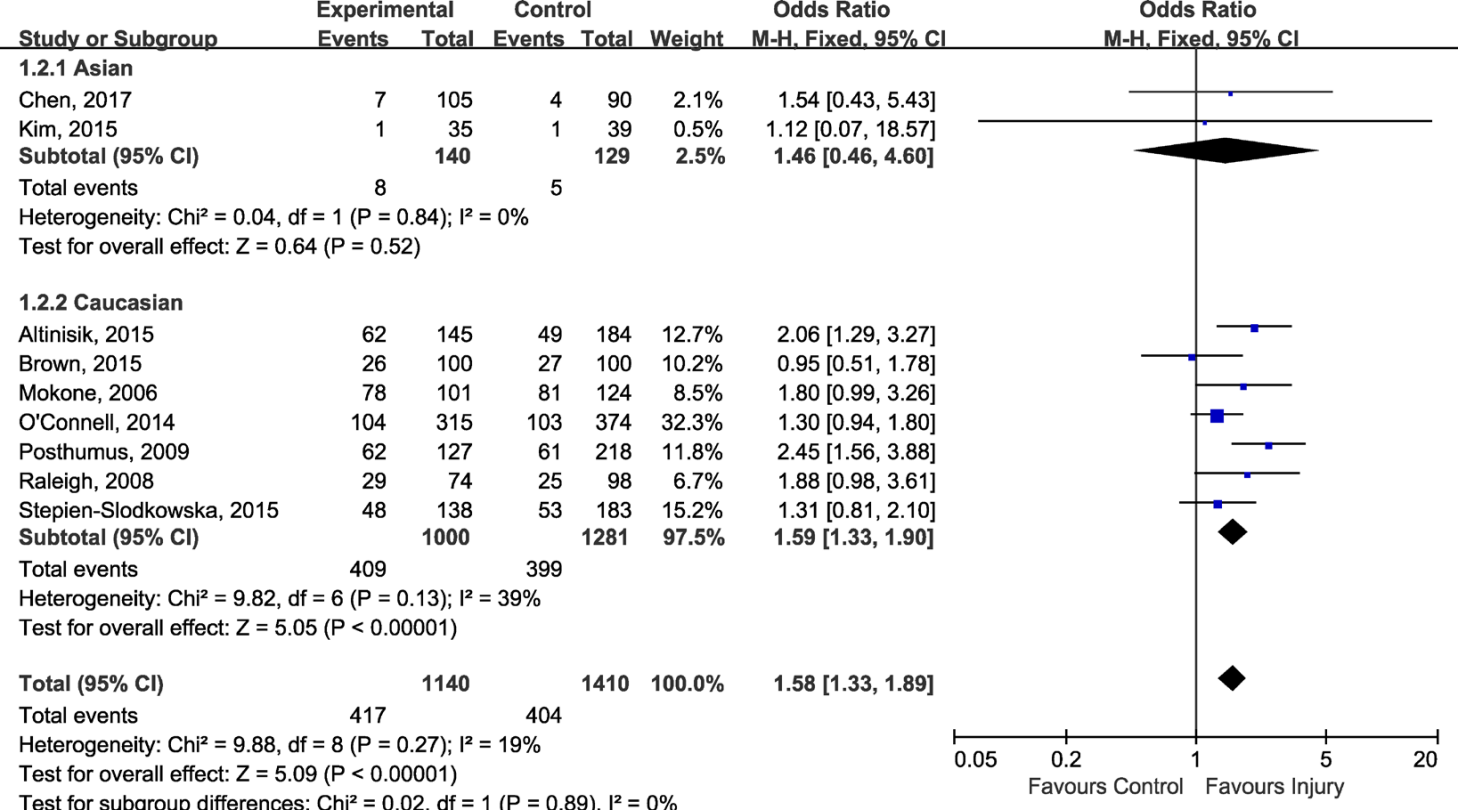
Subgroup-analysis by ethnicity

Although there was no evidence for heterogeneity across included studies, we performed subgroup-analysis according to injury sites to determine whether rs12722 affect different sites of musculoskeletal soft tissue injuries in different ways. The estimated OR between different injury site was similar (test for subgroup differences: *P* = 0.48, I^2^ = 0%), a modest but statistically significant association could be detected in tennis elbow (OR 2.06, 95% CI 1.29, 3.27; *P* = 0.002), Achilles tendon pathology (OR 1.48, 95% CI 1.03, 2.11; *P* = 0.03) and ACL injuries (OR 1.53, 95% CI 1.22, 1.91; *P* = 0.0002) (Figure 3). In order to test whether there was an ethnicity-specific effect we performed the subgroup-analysis by ethnicity, the subgroup analysis (Figure 4) suggested that rs12722 was significantly associated with higher risk of ligament and tendon injuries in Caucasians (OR 1.59, 95% CI 1.33, 1.90; *P* < 0.00001) but not in Asians (OR 1.46, 95% CI 0.46, 4.60; *P* = 0.52). However, the conclusion regarding the association could not be drawn in Asians due to limited number of included studies.

### Sensitivity analysis and publication bias

**Figure 5:**
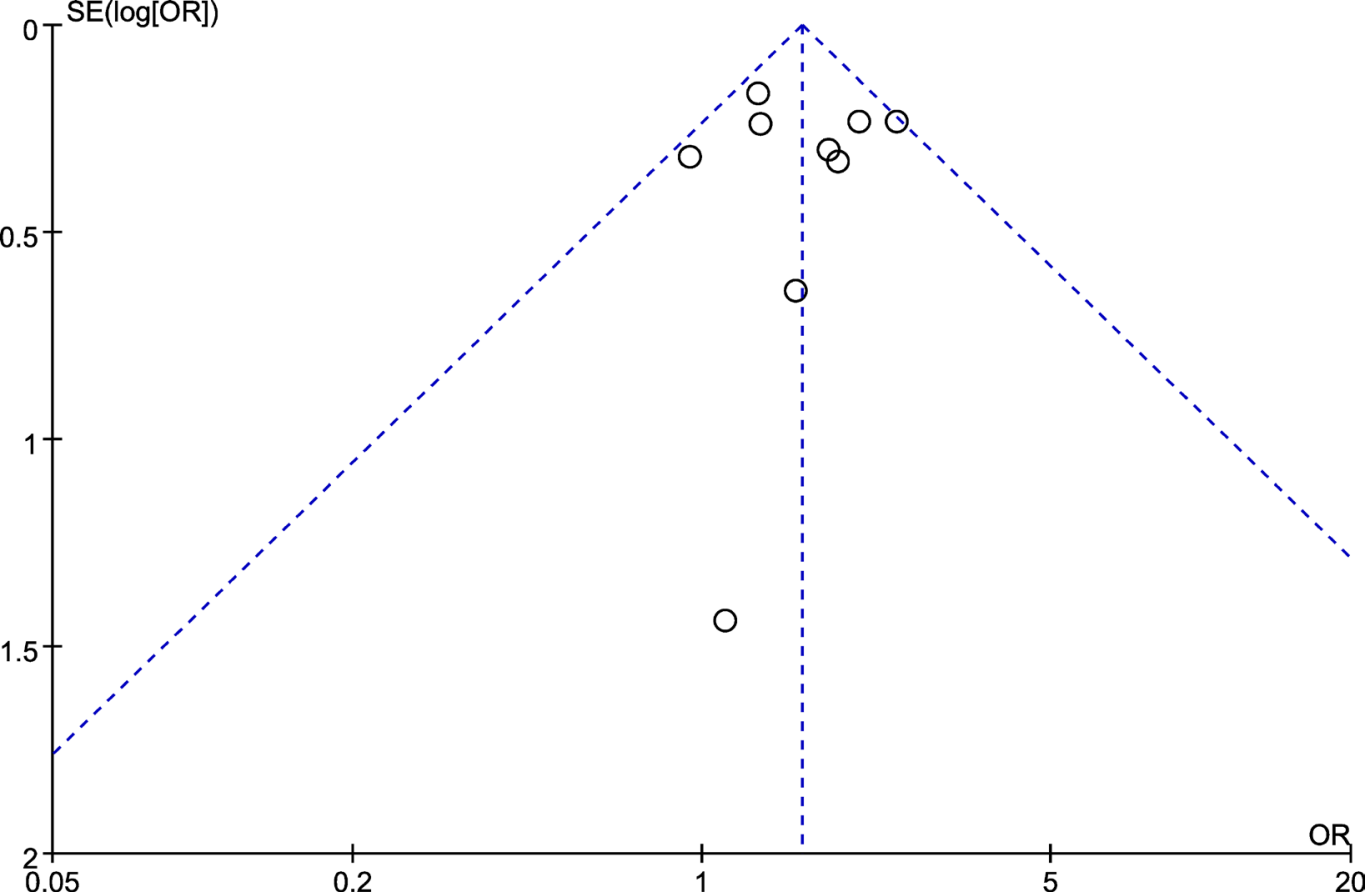
Funnel plot of rs12722 for tendon and ligament injuries

The funnel plot presented symmetry (Figure 5), the Egger’s test (*t* = 0.05, *P* = 0.960) and Begg’s test (*z* = 0.31, *P* = 0.754) also suggested no obvious publication bias. The sensitivity analysis by removing each individual study suggested that the results of our present study were stable (detailed data not shown). In particular, since Mokone et al.’s study [15] did not comply with HWE, we removed this study to test whether the estimated OR was stable, and the results contributed to the robustness of results (OR 1.56, 95% CI 1.30, 1.88; *P* < 0.00001).

## DISCUSSION

Despite the recognition of the accurate risk factors for tendon and ligament injuries may benefit the design and implementation of appropriate therapy interventions in addition to preventive strategies, the precise pathogeny is not yet well ascertained. Intrinsic factors in synergy with extrinsic factors are considered to be the potential etiology. The collagen gene as an intrinsic genetic factor is the focus of various studies in recent years. To the best of our knowledge, this is the first meta-analysis of the association between COL5A1 polymorphism and the risk of tendon and ligament injuries. The main finding of our meta-analysis indicated that individuals with a TT genotype for marker rs12722 were predisposed to an increased risk of musculoskeletal soft tissue injuries in the recessive model. Similar results were detected when the subgroup-analysis was performed by injury sites.

Collagens are a cluster of structural proteins mainly presented in the extracellular space. Vertebrates contain more than 19 different types of collagens and a supplementary 10 collagen-like proteins that comprise a heterogeneous superfamily [29]. They can be briefly classed into fibril-forming collagens, network-forming collagens, beaded filament-forming collagens and the rest. Types I, II, III, V and XI collagens totally belong to fibril-forming collagens, and they have a similar gene structures [29]. They were synthesized and secreted to the extracellular gaps and assembled into fibril architecture in a massive specific tissues such as tendon, ligament, cornea, cartilage, dermis. Type V collagen is a minor collagen that constitutes less than 5% of total fibril collagen content. Although minor in quantity, it is essential for life. Researchers illustrated that COL5A1^−/−^ mice could not survive in utero, as well as COL5A1^+/−^ mice present extremely flawed collagen fibril formation with deficiency of type V collagen reactivity and highly irregular cross-sectional shapes [30].

Type V collagen plays an important role in forming tiny diameter fibrils and appears in tissues where type I collagen is expressed [14]. A previous meta-analysis concluded that the TT genotype of rs1800012 in type I collagen gene was associated with a decreased risk of ligament and tendon injuries [31]. Studies have shown that type V collagen collaborates with type I collagen to generate hybrid fibrils, so as to limit the growth of type I collagen fibrils into thicker fibrils [32, 33]. Type V collagen has several isoforms, most of which contain an alpha chain encoded by COL5A1 gene. The gene has been mapped on chromosome 9q34.2-q34.3, a locus which is associated with tendon injury susceptibility [34]. COL5A1 gene mutations are also strongly linked to classic Ehlers-Danlos syndrome, a severe heritable connective tissue disease presents with laxity and fragility of connective tissues, especially tendons and ligaments [35, 36].

3’-untranslated regions (3’UTR) of mRNAs are of great importance for post-transcriptional regulation. Evidence is mounting to suggest that SNPs including rs12722 located in 3’UTR are involved in many diseases [37]. Rs12722 SNP could affect the secondary structure of 3’UTR. This may influence the stability of COL5A1 mRNA, subsequently results in variation of fibrillogenesis as well as their function and characteristics [38]. In addition, individuals who inherit the haplotype including rs12722 and other loci with modified miRNA recognition site may lead to inaccurate binding of the specific miRNA and translation of COL5A1 [39]. This was potential to influence the proportion of type V and type I collagen expression levels, which was pivotal in impacting fibril strength and diameter. Besides, the C allele represents the wild type sequence appearing more frequent in asymptomatic controls, while the T allele was generally identified in ATP subjects. *In situ* studies also indicated that T functional forms of 3’-UTR might increase mRNA stability and level, thus increasing the production of α-chain, which might change the structure of collagen fibrils [40]. This is consistent with our result that individuals with a TT genotype are more susceptibility to soft tissue injuries. Two of our included studies [23, 27] reported information of SNPs that were in linkage disequilibrium (LD) with rs12722. Stepien-Slodkowska and coworkers [27] reported that rs13946 in COL5A1 was in high LD with rs12722 (D’ = 0.98, r^2^ = 0.52), while Chen et al. [23] replicated this initial finding. However, distinguishing between causal variant and SNPs in LD with the true causal variant is challenging and might require resequencing of candidate region, dense genotyping of all available SNPs. Additionally, further functional studies to confirm the role of COL5A1 gene in ligament and tendon injuries are warranted.

Genetic association studies assessing the risk of a disease relevant to a specific gene polymorphism are often statistically underpowered due to relatively small sample sizes. Thus, evidence synthesis based on multiple molecular association studies could provide a possible solution to this problem by increasing the statistical power. A significant strength of our current study is that we applied a genetic-model free approach to combine evidence instead of assuming the mode of inheritance in advance. Prior to pooling data from individual study, checking for HWE and checking for between-study heterogeneity were made (OR1: I^2^ = 0%, OR2: I^2^ = 44%, OR3: I^2^ = 0%). According to the estimate of the magnitude of effect of a molecular association between rs12722 and musculoskeletal soft tissue injuries, the combined data “dictated” that the recessive model should be assumed for statistical analysis. Since the recessive model was identified as the best-matching genetic model, three genotypes were collapsed into two groups to obtain the pooled results. Before we accepted the estimate of combined data, the between-study heterogeneity was re-checked. Our conclusions were also strengthened by statistically non-significant heterogeneity (I^2^ = 19%, *P* = 0.27). The results indicated a statistically significant association between COL5A1 polymorphism rs12722 and sport-related or occupational-related ligament and tendon injuries, supported by the sensitivity analysis results. Another apparent advantage of the present study is the inclusion of studies from different ethnicities. No language restriction was imposed when establishing the inclusion and exclusion criteria for the retrieval of potentially relevant studies. As a result, seven studies written in English, one study written in Chinese and another in Korean were included for meta-analysis. And the test of publication bias also suggested no statistically significant publication bias among our included studies.

Tendons and ligaments are collagenous tissues with similar composition and hierarchical structures. How the rs12722 polymorphism affects tendon and ligament injuries in different body sites remains unknown. Based on the magnitude of estimate of the association between rs12722 and different injury sites, we cautiously suggest that rs12722 polymorphism in COL5A1 affects the strength and fragility of ligaments and tendons through the same mechanism, evidenced by non-statistically significant subgroup differences (I^2^ = 0%, *P* = 0.48). However, this hypothesis should be confirmed by future studies.

Several limitations to our study shouldn’t be ignored when interpreting the results. First, although we included nine case-control studies in our quantitative analysis, only two of them were conducted within Asian population, rendering the subgroup-analysis by ethnicity almost unfeasible. Future studies focusing on other ethnicities will help validate the molecular association between rs12722 and musculoskeletal soft tissue injury in other populations. Second, both non-modifiable, such as specific polymorphism, and modifiable risk factors including training load are implicated in the etiology of musculoskeletal soft tissue injuries. The cases enrolled in our present study were from different sport groups, and characteristics of different motion groups could lead to an overestimation or underestimation of the drawn conclusion. Thus the association between rs12722 and ligament and tendon injuries could be biased by the above confounder. Third, the mechanism underlying the association we observed remains unknown, additional studies of such molecular mechanisms are needed.

## MATERIALS AND METHODS

This systematic review and meta-analysis was conducted following the Preferred Reporting Items for Systematic Reviews and Meta-Analyses (PRISMA) guidelines.

### Literature search strategy

Five electronic databases including Pubmed, EMBASE, ISI Web of Science, CNKI and Wanfang were systematically searched to identify genetic association studies concerning the polymorphism in COL5A1 gene published before 15 May, 2017. No restriction on language was imposed. A combination of free terms and Medical Subject Headings (MeSH) was used to retrieve all the potentially eligible publications. The search strategy for English databases were as follow: (“Collagen Type V”[Mesh] OR Collagen Type V Alpha I OR Collagen Type V Alpha1 OR Collagen Type 5 Alpha 1 OR Collagen Type V α1 OR Collagen Type 5 α1 OR Type 5 Collagen α1 OR Type V Collagen α1 OR Collagen 5a1 OR COL5A1) and (“Polymorphism, Single Nucleotide”[Mesh] OR Single Nucleotide Polymorphism OR SNP OR SNPs OR mutation OR variation OR polymorphism OR rs12722) and (Injury or injuries or rupture or tendinopathy or dislocation or sprain or strain or muscle tear or Achilles tendon pathology OR Achilles tendinopathy OR Achilles tendon rupture OR Achilles tendon pathologies OR “Anterior Cruciate Ligament Injuries”[Mesh] OR Anterior Cruciate Ligament Injuries OR ACL Injuries OR ACL Injury OR Anterior Cruciate Ligament Injury OR Anterior Cruciate Ligament Tear OR ACL Tears OR ACL Tear OR Anterior Cruciate Ligament Tears OR tennis elbow OR “Tennis Elbow”[Mesh] OR Lateral Epicondylitis). For Chinese academic databanks, we used “ji yin duo tai xing” and “COL5A1” to increase the sensitivity of our literature search. The bibliographic lists of relevant reviews were also manually searched for additional related studies.

### Inclusion and exclusion criteria

Studies satisfying the following criteria were included: (1) enrolled cases should be patients diagnosed as musculoskeletal soft tissue injuries (ACL injury, ATP, Achilles tendon rupture, tennis elbow, and so forth); (2) control groups should consist subject without any history of ligament and tendon injuries; (3) observational studies (case control or cohort studies) on humans that analyzed polymorphism in COL5A1 gene; (4) included articles should report sufficient data to estimate an odds ratio (ORs) with 95% confidence interval (95% CI).

Studies were excluded for following reasons: (1) animal studies, case report or case series, expert opinion; (2) studies failed to provide sufficient data to calculate OR and the associated 95% CI; (3) studies duplicated for retrieval. The most comprehensive study was selected if data reported were duplicated or had been reported for more than once.

### Data extraction and quality assessment

Two investigators (Z. Lv and S. Gao) screened each article independently and were blinded to the findings of the other reviewer. According to the predetermined inclusion criteria, two reviewers conducted a strict screening to identify qualified articles independently, and they extracted data from these eligible papers using a standardized data collection sheet, which included first author, year of the publication, country, ethnicity of enrolled subjects, diagnosis of cases, genotypes distribution of cases and controls, Hardy-Weinberg equilibrium (HWE). Discrepancies between the two reviewers were resolved through discussion until a consensus could be reached. The third review author (P. Cheng) was consulted if a consensus could not be reached.

The Newcastle-Ottawa Scale (NOS) [41] for the assessment of observational studies was employed to assess the methodological quality of eligible studies. Three broad perspectives including selection of cases and controls, comparability of the groups and ascertainment of outcome of interest were evaluated using the Star system (http://www.ohri.ca/programs/clinical_epidemiology/oxford.asp). Two reviewers assessed the methodological qualities of included studies independently, the results of risk of bias judgement were compared afterwards.

### Statistical analysis

The goodness of fit of HWE was assessed using the Chi-square test in control subjects of each included study. The association between rs12722 and musculoskeletal soft tissue injuries was indicated as OR and 95% CI. To determine the most appropriate genetic model for COL5A1 polymorphism in the risk of ligament and tendon injuries, we used a genetic model-free approach [28]. In brief, no prior assumptions regarding the genetic model for meta-analysis were made. OR1, OR2 and OR3 were calculated for genotypes TT versus CC, TC versus CC, TT versus TC for each polymorphism that selected for meta-analysis to capture the magnitude of genetic effect. The best matching genetic model was then identified according to the relationships between the following three pairwise comparisons:

1. Recessive model: if OR1 = OR3 ≠ 1 and OR2 = 1;
2. Dominant model: if OR1 = OR2 ≠ 1 and OR3 = 1;
3. Complete over-dominant model: if OR1 = 1, OR2 = 1/OR3 ≠ 1;
4. Codominant model: if OR1 > OR2 > 1 and OR1 > OR3 > 1, or OR1 < OR2 < 1 and OR1 < OR3 < 1.

Once the best-matching genetic model was identified, three genotypes were collapsed into two groups to obtain the pooled results. The heterogeneity between studies was determined using the *Q*-statistical test and *I*^2^ test. The random-effect model and fixed-effect model were used for data combination in the presence (*P* < 0.1, *I*^2^ > 50%) or absence of heterogeneity (*P* > 0.1, I^2^ < 50% indicates acceptable heterogeneity) respectively. In case of statistically significant heterogeneity across studies, subgroup-analysis by injury sites was performed to test whether rs12722 affects different sites of musculoskeletal soft tissue injuries in different ways, subgroup-analysis by ethnicity was performed to test whether there is an ethnicity-specific effect. Sensitivity analysis was conducted by removing each study at a time and reevaluating the resulting effect on pooled data. Egger’s regression test and Begg’s rank correlation test were employed to estimate the potential publication bias (Stata version 12.0, Stata Corp LP, USA). Funnel plots were generated using RevMan 5.3 software (Copenhagen: The Nordic Cochrane Centre, The Cochrane Collaboration, 2014).

## CONCLUSIONS

Achilles tendon pathology, ACL injuries and tennis elbow along with other musculoskeletal soft tissue injuries are major health concerns affecting different levels of athletes and physically active individuals, making the genetic studies in sport increasingly important. Based on the findings of our current study, the polymorphism rs12722 of COL5A1 was positively associated with tendon or ligament injuries, individuals with TT genotype were predisposed to higher risk of Achilles tendon pathology, ACL injuries and tennis elbow. Genetic risk factors should be taken into account when developing multifactorial models to understand the molecular mechanisms underlying musculoskeletal soft tissue injuries. A further understanding of the relationship between gene variants and sport-related or occupational related soft tissue injuries will help clinicians and athletes to optimize the training programs, and thus effectively reduce the overall risk of injury.

## Abbreviations

ACLI: anterior cruciate ligament injury
ATP: Achilles tendon pathology
TE: tennis elbow
TEN: Achilles tendinopathy
RUP: Achilles tendon rupture
RFLP: restriction fragment length polymorphism
SNP: single nucleotide polymorphism
OR: odds ratio
95% CI: 95% confidence interval
NOS: The Newcastle-Ottawa Scale
